# Hypertension in Sub-Saharan Africa: Burden, Barriers and Priorities for Improving Treatment Outcomes

**DOI:** 10.1161/CIRCRESAHA.124.323889

**Published:** 2025-06-20

**Authors:** Modou Jobe, Serigne Mor Beye, Ngone Diaba Gaye, Mame Madjiguene Ka, Pablo Perel, Alexander D. Perkins, Adama Kane, Andrew M. Prentice, Dike B. Ojji, Lamin E.S. Jaiteh, Anthony O. Etyang, Anoop S.V. Shah, Bamba Gaye

**Affiliations:** 1 Nutrition & Planetary Health Theme, Medical Research Council Unit The Gambia at London School of Hygiene & Tropical Medicine, Banjul (M.J., A.M.P.).; 2 Unité de Formation et de Recherche (UFR) des Sciences de la Santé, Université Gaston Berger de Saint Louis, Senegal (S.M.B., A.K.).; 3 Department of Cardiac Rehabilitation, Ibra Mamadou Wane Medical Center, Dakar, Senegal (N.D.G.).; 4 Department of Cardiology, Principal Hospital of Dakar, Senegal (M.M.K.).; 5 Department of Non-Communicable Disease Epidemiology, London School of Hygiene & Tropical Medicine, United Kingdom (P.P., A.D.P., A.S.V.S.).; 6 Department of Internal Medicine, Faculty of Clinical Sciences, University of Abuja, Nigeria (D.B.O.).; 7 Department of Internal Medicine, Edward Francis Small Teaching Hospital, Banjul, The Gambia (L.E.S.J.).; 8 Epidemiology and Demography Department, Kenya Medical Research Institute (KEMRI)-Wellcome Trust Research Programme, Kilifi, Kenya (A.O.E.).; 9 Department of Cardiology, Imperial College National Health Service (NHS) Trust, London, United Kingdom (A.S.V.S.).; 10 Department of Public Health, Cheikh Anta Diop University, Dakar, Senegal (B.G.).; 11 Alliance for Medical Research in Africa (AMedRA), Dakar, Senegal (B.G.).; 12 Department of Biomedical Informatics, Emory University School of Medicine, Atlanta, GA (B.G.).

**Keywords:** Africa South of the Sahara, cardiovascular diseases, hypertension, myocardial ischemia, renin-angiotensin system

## Abstract

The burden of hypertension is rising rapidly in sub-Saharan Africa (SSA), posing significant health challenges and economic costs that hinder national development. Despite being well-studied in clinical medicine, the detection, treatment, and control of hypertension in SSA remain inadequate. This is due to barriers across the care continuum, including individual-, provider-, and system-level obstacles within the health system. A critical issue is the lack of contextualized mechanistic research to understand the mechanisms, phenotypes, and treatment responses in native SSA populations. Current treatment approaches are often based on data from diaspora Africans, particularly African Americans. Consequently, most guidelines do not recommend angiotensin system drugs as first-line agents for Black patients, a stance that should be reconsidered given some evidence of their effectiveness in native SSA populations. Addressing these barriers requires a comprehensive, multisectoral strategy that includes both preventative and clinical measures at the population and individual levels. Preventative approaches should encompass health and nutrition education, improving food supply quality, and implementing comprehensive transportation and environmental policies. In addition, strategies should be developed to increase the detection of undiagnosed cases through enhanced screening and treatment access to those not receiving care, and revisit current treatment approaches to ensure that they are more tailored to the specific populations and settings. In conclusion, innovative strategies are needed to identify and overcome barriers to hypertension diagnosis and management. A coordinated, multisectoral approach that includes a contextualized mechanistic research agenda, as well as task shifting and task sharing, will help prevent and reduce hypertension in SSA.

Hypertension affects over 1.39 billion people globally, > 75% of whom (1.04 billion people) live in low- and middle-income countries.^1,2^ It is the leading underlying cause of death worldwide, causing an estimated 10 million annual deaths.^3^ Hypertension is generally present in all countries and societies though its prevalence varies significantly. These differences are primarily driven by the lifestyle and environmental factors unique to each population.^4^ The prevalence of hypertension is expected to continue rising due to population aging, increasing sedentary lifestyles, rising obesity rates, and the adoption of unhealthy diets. This trend is likely to escalate the burden of cardiovascular diseases, including stroke, ischemic heart disease, and heart failure, as well as chronic kidney diseases, vision loss, and sexual dysfunction.^5^

While the age-standardized prevalence of hypertension decreased by 2.6% in high-income countries between 2000 and 2010, it increased by 7.7% in low- and middle-income countries.^6^ Furthermore, of 8.5 million deaths attributable to hypertension in 2015, 88% occurred in low- and middle-income countries.^7^ Sub-Saharan Africa (SSA) bears the greatest burden compared with other low- and middle-income regions.^8^ There has been a steady increase in the burden of hypertension in the region, which has increased from 54.6 million in 1990 to 92.3 million in 2000 (70% rise) and 130.2 million in 2010 (41% increase from 2000). It is projected to further affect 216.8 million (66% from 2010) by the year 2030 if appropriate measures are not taken.^9^ Already in the region, hypertension imposes significant direct and indirect economic costs to patients, their families, and national economies.^10–12^ In SSA, hypertension was linked to more than half a million deaths and 10 million years of life lost in 2010 alone. The risk of stroke on the continent also increased by nearly 50% between 1990 and 2015.^13^ In addition to stroke, the burden of coronary heart disease and heart failure has also risen markedly in recent decades, significantly contributing to morbidity and mortality in the region.^14,15^

In this review, we examine the burden of hypertension among African populations in comparison to other groups, explore the key barriers to effective hypertension care in SSA, and discuss potential strategies to address the hypertension burden across the continent.

## Racial and Geographic Disparities

Studies on hypertension have frequently compared Black and non-Black populations, highlighting racial differences and associated health outcomes. It is, however, crucial to note that most of these studies did not include native African populations. Such comparative studies are typically conducted in biracial or multiracial societies, which are uncommon in most parts of SSA.

In the United States, Black populations face a higher risk of hypertension and consistently exhibit elevated blood pressure levels compared with their White counterparts at all ages. Black boys and girls aged 8 to 17 years were found to have 2.9 and 1.6 mm Hg higher systolic blood pressure, respectively, compared with age-matched White boys and girls.^16^ In addition, Black populations experience significantly worse outcomes, including earlier onset of stroke, a 2-fold higher stroke mortality rate, and a 5-fold greater incidence of end-stage renal disease.^17^

The risk of hypertension and its related outcomes is not homogeneous among African-origin populations. A study by Cooper et al^18^ in 7 populations of West African origin describes a consistent gradient in the prevalence of hypertension rising from 10% to 15% in Africa to 20% to 25% in the Caribbean and to 33% in the United States. The Research on Obesity and Diabetes among African Migrants (RODAM) study compared hypertension prevalence, awareness, and control among Ghanaians (all born in Ghana), including those living in Ghana and various European cities. The study found differences in outcomes, with better results in those living in Europe.^19^ These findings suggest that the causes contributing to the racial and geographic disparities (among Black populations) are multifactorial, including genetic, lifestyle, and environmental factors, as well as access to quality health care.^20^

## Genetic Factors and Environmental Influences

Hypertension in SSA is influenced by a complex interplay of genetic factors and environmental influences. Understanding this interaction is critical for developing effective prevention and treatment strategies tailored to the region. SSA is one of the most genetically diverse regions globally.^21^ Variants in candidate genes such as *ACE*, *AGT*, and *NOS3* have been associated with hypertension in African populations.^22^ In addition, some populations of African origin carry genetic variants that increase salt sensitivity. For example, variants in the *SLC4A5* gene, which regulates sodium transport, have been linked to salt-sensitive hypertension.^23^ Furthermore, the *APOL1* gene, which is more prevalent in populations of African origin, is associated with kidney disease and may contribute to hypertension-related complications. However, it is important to note that genetic predisposition alone is rarely sufficient to cause hypertension or increase its risk without environmental triggers such as dietary and lifestyle factors, socioeconomic conditions, and psychosocial stress. This highlights the importance of addressing these environmental factors to effectively prevent and manage hypertension in SSA.

## Role of Race in Antihypertensive Therapy

Although hypertension is one of the most extensively studied diseases in clinical medicine, much of our current understanding of its etiological drivers, resultant phenotypes, and treatment approaches in native Black Africans is derived from studies in diaspora Africans, particularly African Americans.^18,24^ Extrapolating these findings to native Africans may not be justified. This is due to differences in cardiovascular risk profiles, socioeconomic status, and responses to antihypertensive treatment between African Americans and other Black populations, especially native Africans.^25^ Critically, studies on drug responses in hypertension are lacking in SSA. We are aware of only 2 multicountry studies conducted exclusively on sub-Saharan African populations. These are the NOAAH trial^26^ (Newer versus Older Antihypertensive Agents in African Hypertensive Patients) and the CREOLE clinical trials (Comparison of Three Combination Therapies in Lowering Blood Pressure in Black Africans).^27^ The NOAAH trial found a combination of amlodipine/valsartan to be more effective at controlling systolic blood pressure compared with bisoprolol/hydrochlorothiazide in native Africans. In the CREOLE study, amlodipine plus either hydrochlorothiazide or perindopril was found to be more effective than perindopril plus hydrochlorothiazide at lowering blood pressure at 6 months.

The appropriateness of using the Black race to guide the choice of initial antihypertensive therapy, therefore, warrants careful consideration. While practice guidelines^28,29^ favor calcium-channel blockers and diuretics for Black adults with hypertension, these are typically recommended as part of combination therapy with a renin-angiotensin system (RAS) blocker rather than as monotherapy. Importantly, race-specific differences in average blood pressure responses do not reliably predict individual responses to single-drug therapy, as the BP response distributions overlap significantly between Black and White adults.^30^ Therefore, there is no clear advantage to using race as a determinant for selecting single-drug therapy in patients for whom monotherapy is otherwise appropriate.

The Kaiser Permanente’s Gardena Medical Offices in the United States implemented a comprehensive hypertension control program to address racial disparities in blood pressure management. The program that included a patient population that was 65% Black utilized a team-based approach, culturally tailored communication, physician-led initiatives to reduce therapeutic inertia, and adherence to evidence-based guidelines.^31^ A race-agnostic therapeutic algorithm was also introduced to guide treatment decisions. The program initially aimed to close the racial gap in blood pressure control (<140/90 mm Hg), which stood at 6.3% (76.6% control in Black patients versus 82.9% in others). Over several years, blood pressure control rates among Black patients improved to 81.4%, reducing the disparity to 2.8%. This demonstrated that high blood pressure control rates and reduced racial disparities can be achieved without race-specific prescribing, particularly when combined with intensive, guideline-aligned care and efforts to avoid therapeutic inertia.

### Lifestyle Approaches in Hypertension

As hypertension care moves away from race-based prescribing, race-informed approaches are increasingly focusing on dietary and lifestyle changes. Practice guidelines^28,29^ highlight nonpharmacological strategies, such as weight loss, increased physical activity, limited alcohol intake, and dietary modifications (eg, reduced sodium, increased potassium, and adoption of a DASH-like diet). While these strategies benefit adults of all races, evidence suggests they may be particularly effective for Black patients, who often experience greater blood pressure reductions from dietary interventions.

Black adults tend to consume higher levels of sodium and lower levels of potassium compared with their White counterparts,^32^ which exacerbates hypertension risks. Increasing potassium intake, especially in the context of high sodium consumption, can significantly lower blood pressure and reduce salt sensitivity. The Dietary Approaches to Stop Hypertension (DASH) diet, rich in fruits, vegetables, low-fat dairy, whole grains, and lean proteins, has been shown to produce twice the blood pressure–lowering effect in Black adults compared with White adults.^33^

Physical activity also plays a critical role in hypertension management. While beneficial for all races, Black adults are less likely to engage in moderate-to-vigorous physical activity, increasing their risk of hypertension.^34^ Encouraging increased physical activity in Black patients is likely to yield significant health benefits.

Lifestyle factors such as unhealthy diet and physical inactivity are strongly linked to obesity, which, in turn, is a major risk factor for hypertension in SSA. Findings from the H3Africa (Human Hereditary and Health in Africa)CHAIR (Cardiovascular H3Africa Innovation Resource) study, involving 30 044 participants across 13 African countries, demonstrated a consistent association between obesity and elevated rates of hypertension.^35^ This aligns with numerous other studies on the continent,^36,37^ further emphasizing the critical role of obesity in the region’s hypertension burden. Race-informed dietary and lifestyle interventions, particularly those emphasizing potassium-rich diets and increased physical activity, should, therefore, be prioritized for Black adults with hypertension to achieve better health outcomes.

## The Higher Burden of Hypertension in Black POPULATIONS

Several theories have been advanced to explain possible reasons behind the higher burden of hypertension among African-origin populations. However, it must be emphasized that these theories are mostly based on diaspora Africans and may not be applicable, at least universally, to native sub-Saharan Africans. Some of the theories are highlighted in the following.

### Primary Aldosteronism Is Common in Black Populations

Primary aldosteronism (PA) is a common cause of secondary hypertension, characterized by excessive aldosterone production leading to sodium retention, hypokalemia, and increased blood pressure. It is an independent risk factor for cardiovascular, renal, and metabolic diseases, whose early detection and targeted therapy could prevent complications in many patients with hypertension.^38^ It has been previously underdiagnosed, especially in Black populations, but emerging evidence suggests that its prevalence may be higher than previously thought. In a prospective study of 88 consecutive patients referred to a specialist clinic for resistant hypertension, PA was present in 20% of patients, and the prevalence was similar between Black (23%) and White (18%).^39^ These data are supported by data from the SABHA study (South African Burden of Hypertension Assessment), where a high prevalence of PA was identified in Black men in South Africa.^40^ The genetics of PA in Black patients with hypertension is complex and differs in some important ways from other populations. A study by Nanba et al^41^ found that somatic CACNA1D (Calcium Voltage-Gated Channel Subunit Alpha1 D) mutations were the most prevalent genetic alteration in Black patients with aldosterone-producing adenomas, followed by KCNJ5 mutations. This contrasts with European and East Asian populations, where KCNJ5 mutations are more common. Black individuals may also have a higher prevalence of bilateral adrenal hyperplasia, a subtype of PA, which may have a distinct genetic basis compared with unilateral aldosterone-producing adenomas. This may have important implications for their management, as medical therapy instead of surgery is preferred in those with bilateral adrenal hyperplasia.^42^

### Higher Salt Sensitivity and Salt Retention in Black Patients With Hypertension

Salt sensitivity plays a crucial role in the development of hypertension, especially among Black patients, in whom it is more pronounced compared with White individuals. Studies consistently show that Black individuals exhibit higher rates of salt sensitivity. For example, a crossover trial comparing ethnic differences in salt sensitivity found that hypertensive Black women experienced a greater increase in mean arterial pressure (12.6 versus 8.2 mm Hg) following high-salt intake, indicating a heightened sensitivity to sodium-induced blood pressure elevation.^43^ In addition, a study in Nigeria demonstrated that pressure responses to acute salt loading were significantly higher in hypertensive (60.7%) compared with normotensive individuals (52.0%).^44^ Several factors including genetic, epigenetic, environmental/social determinants, and diet have been implicated in renal, neural, and vascular mechanisms leading to salt-sensitive hypertension in Black patients.^45^ A leading hypothesis for the increased salt sensitivity is through mutations in the ENaC (epithelial sodium channel),^46^ which regulates sodium reabsorption in renal tubules. Furthermore, Black individuals, particularly women, exhibit increased oxidative stress and inflammation, which can impair endothelial function and exacerbate blood pressure elevation in response to salt intake.^47^

### Liddle Phenotype Tends To Be Higher in Black Populations

Liddle syndrome is a rare, monogenic form of hypertension characterized by excessive sodium reabsorption, hypokalemia, and low levels of renin and aldosterone despite elevated blood pressure levels. Although initially described in White populations, recent studies have highlighted a higher prevalence in individuals of African descent. In a study in South Africa, Black individuals were found to have significantly lower renin and aldosterone levels compared with their White counterparts, suggesting a genetic predisposition to sodium retention. Studies in South Africa have identified specific mutations in the *SCNN1B* gene, which encodes the β-subunit of ENaC, associated with Liddle syndrome.^42^ The p.Arg563Gln (R563Q) mutation was found in 5.9% of hypertensive Black South Africans compared with 1.7% in normotensive individuals, suggesting a strong association with hypertension in this group.^48^ This mutation was, however, notably absent in West African populations.

A recent study investigating hypertension in 3 African nations (Nigeria, Kenya, and South Africa) analyzed candidate genes linked to the Liddle phenotype in 14 individuals. The researchers identified 4 nonsynonymous variants in the *GRK4* gene (*R65L*, *A116T*, *A142V*, and *V486A*), with each patient carrying at least one. Notably, 3 of these variants had been previously linked to hypertension. The *SCNN1B* gene revealed 3 nonsynonymous variants (*R206Q*, *G442V*, and *R563Q*), 2 of which were previously known, with one associated with hypertension. The *NPPA* gene showed a novel nonsynonymous variant (*V32M*), while no variants were found in *NEDD4L*. Finally, the *UMOD* gene presented 3 nonsynonymous variants: *D25G*, *L180V*, and *T585I*.^49^

### HIV-Associated Hypertension

HIV remains a significant public health challenge in SSA, particularly in Eastern Africa and Southern Africa. The intersection of HIV and hypertension warrants greater attention, as their co-occurrence poses unique challenges for health care systems and patient outcomes. The mechanisms of hypertension in people living with HIV are complex and multifactorial. With effective antiretroviral therapy, people living with HIV are living longer, increasing the prevalence of age-related hypertension.^50^ Some factors are HIV specific, and others are related to the effect of the antiretroviral therapy. During the asymptomatic phase, CD4+ cells decline, leading to gut dysbiosis and bacterial translocation, which exacerbate inflammation. This triggers inflammatory cytokines (eg, interleukin [IL]-17 and IL-6) and T-cell infiltration into organs such as the kidneys and liver, activating the RAS.^51^ RAS that can also be activated by drugs, particularly by protease inhibitors, increases blood pressure through vasoconstriction, sodium retention, and aldosterone release.^52^ In addition, ART-induced lipodystrophy and adipocyte proliferation promote macrophage infiltration, adipokine release, and insulin resistance, further impairing nitric oxide production, thereby leading to vasoconstriction and elevation of blood pressure.^53^

Chronic HIV infection begins with dendritic cells in the gastric-associated lymphoid tissue, which activate CD4+T cells. These, in turn, stimulate CD8+T cells, leading to arterial stiffness. Activated arterial endothelial cells promote the expression of adhesion molecules (intercellular Adhesion molecule [ICAM], vascular cell adhesion molecule [VCAM], and Platelet-derived growth factors [PDGF]) facilitating leukocyte adhesion to arterial walls and contributing to atherosclerosis.^54^ Advanced infection involves HIV-1 glycoprotein 120 (gp120) increasing ET1 (endothelin-1) and reducing nitric oxide, causing vasoconstriction.^55^ Furthermore, antiretrovital therapy (ART) and lifestyle changes in HIV-infected patients, such as central obesity, smoking, physical inactivity, and excessive alcohol intake also influence nitric oxide levels.^54^

## Barriers to Hypertension Management

**Figure 1.**
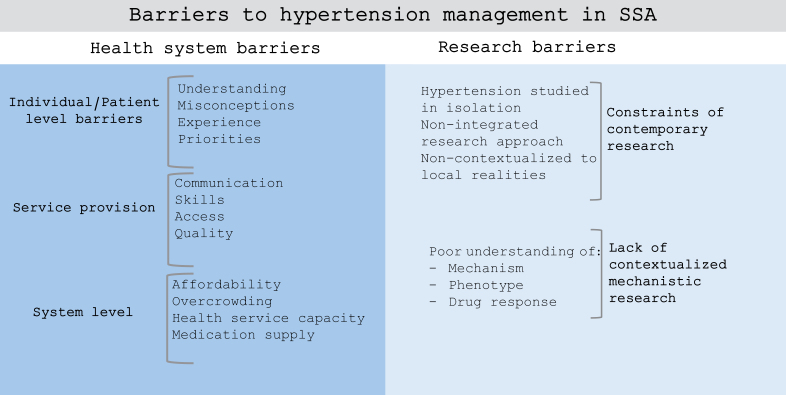
Barriers to hypertension management in sub-Saharan Africa (SSA).

The barriers to effective hypertension management in SSA are multiple and complex. Besides the health system barriers, there is also a lack of contextualized mechanistic research (Figure 1). Although there are concerns and growing calls to move away from race-based prescribing, this does not negate the need for further research to understand hypertension mechanisms, phenotypes, and drug responses in SSA, which are crucial to improving outcomes.

### Health System Barriers

To significantly reduce hypertension-related burden, there is a need to strengthen the entire hypertension care cascade from awareness (through screening and diagnosis) to treatment (including risk stratification and appropriate initiation of treatment) and ultimately to long-term control (which includes regular monitoring, ensuring medication adherence, and timely referral for specialist care if required).^56^ The health system barriers to improving hypertension care occur at various levels impeding effective hypertension prevention, diagnosis, and management. These barriers are briefly discussed in the following.

#### Individual-Level Barriers

A major challenge often arises from the asymptomatic nature of hypertension. Many individuals are unaware of their hypertensive status until serious complications such as heart failure, stroke, or kidney disease occur. This lack of awareness is exacerbated by low health literacy, competing priorities such as income-generating activities or family obligations. In some settings, traditional health beliefs or spiritual interpretations of illness lead individuals to delay or avoid seeking care, instead turning to traditional healers or home remedies.^57–60^ These barriers are compounded by misconceptions about the cause of hypertension and the potential benefits of drug treatment. These perceptions highlight the need for community-based health education campaigns that not only raise awareness but also tackle prevailing myths and misconceptions.

#### Provider-Level Barriers

One of the major challenges is the lack of adequately trained health care personnel, particularly in primary care and rural settings. Many health workers in SSA lack the training to accurately diagnose, risk-stratify, and manage hypertension according to current guidelines. Communication between health care workers and their patients is often sub-optimal. This, among others, leads to poor patient engagement with the health care system. In addition, some health facilities frequently lack sufficient basic resources, such as calibrated blood pressure machines, or laboratory services required for proper diagnosis, assessment, and monitoring.^60^

#### System-Level Barriers

Several structural issues hamper the delivery of consistent, high-quality hypertension care. Access to health services is unevenly distributed, with rural and periurban populations facing difficulties due to long travel distances, poor transport infrastructure, and a shortage of health facilities. Overcrowding in urban public health clinics often results in long waiting times and short consultation durations, further deterring patients from seeking care unless symptoms are severe. In addition, health facilities across the continent frequently face limited, inconsistent, or insufficient supplies of antihypertensive medications. Even when treatment is available, it is often unaffordable, exacerbated by inadequate coverage from national health insurance schemes. Addressing these challenges requires urgent attention to the underinvestment in health care service capacity.^61^

### Research Barriers

#### Constraints of Contemporary Research Approaches

There has been a lot of research on hypertension globally and, to some extent, in SSA. In the region especially, the current research approaches have 3 major limitations.

First, much of the research conducted to address noncommunicable diseases focuses on individual cardiovascular risk factors. However, hypertension rarely exists in isolation; it often coexists with other conditions such as diabetes, obesity, and dyslipidemia. This narrow research approach also overlooks the international consensus for an overall CVD risk assessment, which considers multiple cardiometabolic risk factors to guide risk evaluation and management.^62^ Cardiovascular risk stratification in SSA requires tools and approaches that are simple, affordable, and tailored to low-income settings. The steps to developing such tools are, however, beyond the scope of this review. A few considerations are, nevertheless, worth mentioning. As the region often faces challenges such as limited health care infrastructure and a lack of diagnostic resources, easily measurable tools such as age, sex, blood pressure, and smoking status should be used to develop such tools. An example of this is the nonlaboratory-based World Health Organization (WHO)/International Society of Hypertension (ISH) Risk Prediction models.^63^ Although useful, it has limitations such as excluding individuals under 40 years of age. Risk assessment tools should be developed using local data on prevalent risk factors and should account for the impact of infectious diseases and undernutrition on cardiovascular risk. In addition, mobile-based applications or digital tools that calculate region-specific risk scores should be developed to ensure ease of use and accessibility.

Second, existing research has predominantly focused on isolated implementation strategies at the facility or provider level. However, lessons from managing other chronic diseases, such as HIV in SSA, underscore the importance of adopting multifaceted, community-based approaches.^64^ Initiatives such as the Addressing Cardiovascular Health Inequities in Emerging Settings and Populations project seek to address this gap in SSA.^65^ This initiative introduces an innovative, ecosystem-driven strategy to tackle hypertension in Africa. The approach employs an iterative implementation cycle, focusing on creating and deploying context-specific solutions that address barriers and enhance facilitators. Central to this strategy is ensuring effective communication and active participation from all stakeholders involved in the implementation process.^66^

Finally, studies to date have failed to consider the broader health system context, hindering scale-up and sustainability. A contextualized community-based program that addresses overall cardiovascular disease risk through a combination of strategies has the potential to significantly improve cardiovascular disease outcomes in SSA, but evidence to guide such an approach is lacking. This is discussed in more detail in the following.

### Lack of Contextualized Mechanistic Research

There has been little research on the mechanisms, phenotypes, and drug response in SSA to understand and improve treatment outcomes in SSA. Most of such research in diasporan Africans has largely focused on the RAS. However, the role of the systemic RAS in the pathophysiology of hypertension in Black populations especially native Africans has been questioned. There are reports, mainly from diasporan Africans, of significantly lower levels of plasma renin in Black populations compared with White independent of age and blood pressure status.^67–69^ Reports from Southern Africa show a lack of difference in renin levels in normotensive and hypertensive Black patients, further supporting the theory of low renin levels as being a feature in Black populations.^70^ The low plasma renin levels in Black populations are attributed to their higher sodium retention and consequent decrease of renin release from the juxtaglomerular apparatus and suppression of the systemic RAS^71,72^ (a phenotype that may have been selected among survivors of the slave trade sea crossings and that may, therefore, differ between diasporan and native Black Africans). This has major treatment implications such that drugs blocking the RAS are not recommended as monotherapy in Black patients in the absence of other compelling indications.^73^ This treatment recommendation, however, is still a subject of controversy.^74^

**Figure 2.**
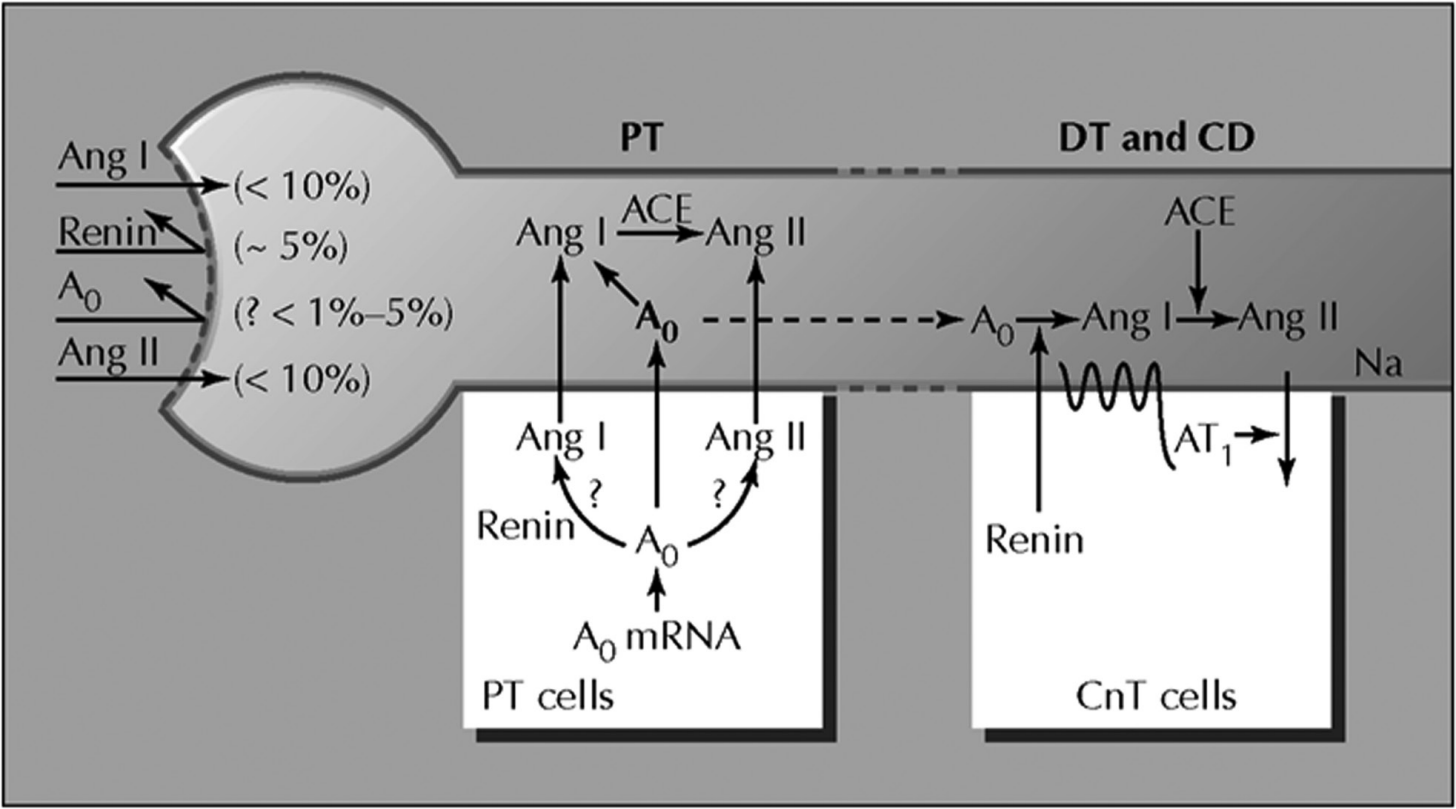
**Tubular renin-angiotensin system in proximal and distal nephron segments.** In Ang (angiotensin) II hypertension, increased proximal tubular secretion of Ao (angiotensinogen) spills over into the distal nephron and increases Ang II effects on distal tubular reabsorption. ACE indicates angiotensin-converting enzyme; CD, collecting duct; CnT, connecting tubule; DT, distal tubule; and PT, proximal tubule. Reproduced from Navar et al^76^ with permission from Springer Nature.

The low plasma renin levels found in Black individuals may be paradoxical and misleading as the RAS may truly be activated at the tissue level in the kidney, which would then feedback via high sodium retention to suppress plasma renin levels.^75^ The main sites of intrarenal RAS activation are the proximal and distal tubule and collecting duct segments of the nephron. As depicted in Figure 2, almost none of the plasma angiotensinogen and renin and little circulating angiotensin I and II (<10%) are filtered across the glomeruli.^76,77^ However, the proximal tubule secretes angiotensinogen, which is converted to angiotensin I and subsequently angiotensin II through the actions of intrarenally derived renin- and angiotensin-converting enzymes. Both are also detected in the distal nephron and with spillover of angiotensinogen distally; this leads to enhanced angiotensin II production. Angiotensin II in the distal nephron (1) stimulates luminal AT1 (angiotensin II receptor type 1) receptors leading to enhanced sodium reabsorption and (2) synergistically enhances proximal sodium reabsorption rate. All these together with the effect of aldosterone result in the elevation of blood pressure. Angiotensin II also exerts a positive feedback action on intrarenal angiotensinogen mRNA and protein (therefore, resulting in higher angiotensinogen concentrations).^77,78^ As a result, the angiotensinogen detected in the urine reflects overall kidney function and intrarenal RAS activity.

Despite the strong evidence for intrarenal activation as a driver of hypertension in Black people, it has not been comprehensively studied in African populations. There have only been 2 reports in African populations to the best of our knowledge. The first included a small sample size of 12 African men.^79^ The other study, which included a larger sample size, predominantly included women and subjects with obesity, thus limiting its generalizability.^80^ It is, therefore, crucial to conduct research on the RAS, which may offer valuable insights and improve treatment outcomes.

Besides research on the RAS, there is also a lack of data on PA in SSA. Most of the available evidence from the continent is from Southern Africa. This may not represent the genetic diversity and environmental influences on the continent. Furthermore, more investment in diagnostic infrastructure is also required to provide access to sensitive assays for renin and aldosterone, which are mostly unavailable in many settings in Africa.

## Addressing the Barriers: Practice and Policy Approaches

In the following, we discuss various practice and policy approaches to address the hypertension burden in SSA. These strategies encompass both preventive and clinical measures, each of which should be carefully considered.

### Preventative

The high prevalence of hypertension underscores the need for a coordinated, multisectoral approach to comprehensive noncommunicable disease prevention and control programs. Currently, population-wide strategies for preventing hypertension and other noncommunicable diseases are insufficient. Many existing programs in SSA remain predominantly clinical rather than preventative, and where preventative initiatives do exist, they are often limited to a few municipalities rather than being implemented on a broader, population-wide scale.^81^ Without such intervention, the region’s underresourced health systems are likely to face a significant burden of complications from target organ damage, including stroke, ischemic heart disease, and chronic kidney disease. These complications often affect the most productive age groups, leading to greater economic burden to individuals, their families and national economies, and premature mortality. A strategy developed using a participatory approach and reaching all sectors of society is urgently needed. A coordinated multipronged prevention program addressing 3 main areas should be considered.

1. Health and nutrition education and promotion: These programs have been shown to be effective in reducing the burden of noncommunicable diseases, including hypertension,^81–83^ and should be adapted and embedded in school programs, workplaces, health facilities, and into community activities to achieve the maximum effect. These health programs could be delivered through electronic media (radio and television), print media (eg, newspapers), and other platforms. They should be led by relevant units within ministries of health, other government departments, and development partners. The World Health Organization recognizes and promotes this approach.^84^

This approach will empower individuals and communities with the knowledge needed to adopt healthier lifestyles, including understanding hypertension, its risk factors, and its consequences. It will also raise awareness about the importance of blood pressure screening for early detection and timely management. While not exhaustive, such programs should encourage healthy dietary habits, such as reducing salt intake, increasing consumption of potassium-rich foods, minimizing trans fats, and promoting physical activity. Importantly, these programs should be tailored to meet the specific needs of different populations. By addressing modifiable risk factors, these initiatives have the potential to significantly reduce the incidence of hypertension, help individuals better manage their condition, and reduce disparities in hypertension prevalence and outcomes, particularly among underserved and high-risk populations.^85^

2. Improve quality of food supply: This involves developing and implementing policies to enhance the processing and manufacturing of foods, increasing their availability and affordability. This critical public health strategy tackles key dietary risk factors for high blood pressure by ensuring access to healthier food options and reducing the availability of unhealthy foods. Countries should regulate or restrict the marketing of unhealthy foods, particularly to children, and implement front-of-package labeling systems, such as traffic light labels, to help consumers easily identify healthier choices. These labels should be designed to be clear and accessible, even to individuals with limited literacy. Efforts to increase the availability of fresh fruits and vegetables, such as supporting community gardens, are essential, especially in SSA, where they provide affordable, locally grown produce. In addition, taxing sugary drinks and other unhealthy products can effectively reduce their consumption.^86^ Such measures have proven effective in lowering hypertension rates and preventing other cardiometabolic diseases.^87^
3. Transportation policy and environmental design: Countries in SSA have undergone rapid unplanned urbanization in recent years. Urbanization is strongly linked to hypertension and other cardiometabolic diseases. To address this, governments should implement robust policies to promote health-focused urban design. This includes creating environments that limit automobile dependency, encourage walking and cycling, and enhance public safety.^88^ This can play a transformative role in improving cardiovascular health. By promoting active transportation, such as walking and cycling, and ensuring access to recreational spaces, communities can encourage regular physical activity. This not only helps lower blood pressure but also strengthens overall cardiovascular health. At the same time, creating quieter, greener, and more walkable environments can significantly reduce stress levels, which are a known contributor to hypertension. These calming spaces offer a respite from the hustle and bustle of urban life, fostering mental and physical well-being.^88^

### Clinical

1. Enhance screening: To the best of our knowledge, there is no formal policy for population-wide hypertension screening in SSA. Although opportunistic screening occurs during consultations in most health care settings,^89^ this is not always available due to a lack of resources (eg, blood pressure monitors).^90,91^ Blood pressure monitors should be made available in health care facilities to facilitate mass screening for both patients and healthy individuals seeking blood pressure checks. Evidence suggests that screening programs in nontraditional settings such as schools, places of worship (eg, mosques and churches), markets, and barbershops complement clinical consultations and serve as an effective strategy for identifying risk factors, detecting undiagnosed cases, and initiating appropriate treatment and long-term management.^92^ A 3-year, community-based intervention program in Tunisia, which promoted physical activity, healthy dietary habits, and tobacco cessation, led to significant reductions in blood pressure and a decline in hypertension prevalence at the population level.^93^ Similarly, in Ethiopia, health extension workers were trained as part of the multicomponent Health Extension Program to actively screen individuals in their communities to detect hypertension early.^94^ These trained health extension workers were found to be as effective in detecting high blood pressure as trained health personnel.^95^ Besides, this strategy has helped to overcome barriers such as distance and transportation costs to health facilities.^96^ Such scalable programs could be adapted to other contexts in SSA. There are currently other initiatives to use community health workers and village health workers to improve detection and management of hypertension in SSA.^97,98^

Current evidence indicates that hypertension is highly prevalent across the continent and is increasingly affecting younger populations. Implementing a policy for periodic screening, including younger individuals, should be considered. This will require training and recruiting more health care personnel, as well as adopting task-shifting strategies to improve access to hypertension diagnosis and management.

2. Increase treatment access: Access to treatment should be improved to ensure that untreated patients receive treatment. As mentioned above, several barriers exist at both the individual and system levels. At the individual level, these include a lack of understanding about hypertension, fear of treatment, and reluctance to take medication, especially when symptoms are absent.^99^ These challenges highlight the need for education on hypertension and the benefits of treatment. At the health system level, barriers include health care workers’ limited understanding of guidelines or their reluctance to follow them. It is, therefore, essential to establish policies and training programs to ensure that health care workers adhere to standardized treatment protocols. In addition, training and recruitment of more health care personnel, as well as adopting task-shifting approaches to increase access to diagnosis and management of hypertension, are required. These approaches should be carefully planned and implemented with key considerations to ensure their success and sustainability. Engaging local communities is crucial, as it raises awareness and fosters their support and active participation.^100^ In addition, such programs should be integrated into existing health systems or structures, such as primary health care clinics and HIV clinics, to facilitate scalability and long-term success, as has been demonstrated in Uganda and Tanzania.^101^ This integration must be complemented by a strong commitment from health authorities and partner institutions to ensure that low-cost or free antihypertensive medications are consistently available and accessible to patients. However, this remains a significant challenge in many sub-Saharan African settings, where there are limited resources, reliance on donor funding, and competing health priorities. Success with this approach has been reported in other settings. A cluster-randomized controlled trial was conducted in rural districts in Bangladesh, Pakistan, and Sri Lanka to address poor hypertension treatment outcomes and control. The intervention, comprising mainly home blood pressure monitoring and counseling, was performed by trained government community health workers linked with public health care infrastructure and led to a greater reduction in blood pressure.^102^
3. Revisiting current treatment approaches: This requires a multifaceted approach, including the development of comprehensive strategies to strengthen support for patients and their caregivers. Adherence to treatment must be reinforced among those currently receiving care. Given that many patients receiving treatment fail to achieve optimal outcomes, current pharmacological treatment guidelines should be revisited to develop more effective therapies tailored to native African populations. Most guidelines recommend a combination of thiazide diuretics and calcium-channel blockers as first-line agents for Black patients, as these have been reported to be more effective than renin-angiotensin blockers.^103^ However, such research is now being contested. Future research should also systematically evaluate health service and system structures, strengthen the evidence base for identifying individuals who would benefit most from treatment, and develop more effective risk stratification approaches suitable for low-income settings.

The WHO HEARTS technical package offers evidence-based guidelines for hypertension management in low-resource settings.^104^ Adapting these strategies to SSA requires addressing the region’s unique challenges. These include limited health care infrastructure, which was primarily designed for managing communicable diseases, conditions that often do not require long-term follow-up. To ensure scalability and sustainability, task shifting and capacity building should be prioritized, and hypertension care should be integrated into existing health systems.

## Conclusions

The current state of hypertension in SSA is a public health concern requiring urgent attention. Tangible and innovative research approaches are required to urgently address the rising trend and identify barriers to management, thereby improving treatment outcomes. A coordinated multisectoral approach including task shifting and task sharing will significantly improve prevention and increase case detection, treatment allocation, and outcomes. Finally, research should be streamlined and adapted to respond to priorities in SSA.

## Article Information

### Sources of Funding

None.

### Disclosures

None.
